# Association Between Loneliness and Hikikomori Symptoms. A Systematic Review and Meta‐Analysis

**DOI:** 10.1002/mpr.70095

**Published:** 2026-08-03

**Authors:** André Hajek, Hans‐Helmut König

**Affiliations:** ^1^ Department of Health Economics and Health Services Research University Medical Center Hamburg‐Eppendorf Hamburg Center for Health Economics Hamburg Germany

**Keywords:** hikikomori, loneliness, prolonged social withdrawal, psychometric meta‐analysis, social exclusion, social isolation, systematic review

## Abstract

**Objectives:**

A systematic review and meta‐analysis were conducted to investigate the association between loneliness and hikikomori symptoms.

**Methods:**

We searched three electronic databases (plus hand search). Our review followed the PRISMA guidelines, and a PROSPERO registration was made. We included observational studies determining the association between loneliness and hikikomori symptoms.

**Results:**

Eight cross‐sectional studies were finally included. Our psychometric meta‐analysis revealed a mean corrected correlation of 0.68 (95% CI: 0.62 to 0.74). The mean corrected correlations of loneliness with the subscales socialization, isolation, and emotional support were 0.54 (95% CI: 0.50 to 0.58), 0.62 (95% CI: 0.60 to 0.64), and 0.71 (95% CI: 0.67 to 0.75), respectively. The mean corrected correlation for the association between loneliness and hikikomori symptoms was 0.66 (95% CI: 0.62 to 0.70) among European countries, whereas it was 0.90 (95% CI: 0.81 to 0.99) among Asian countries. Five of the studies were of good quality, and three were of fair quality.

**Conclusion:**

Our work identified a strong, positive association between loneliness and hikikomori symptoms. Upcoming longitudinal studies are required to clarify the directionality between such factors. There is also a clear need for research in regions that have been neglected so far.

## 1 Introduction

Hikikomori refers to a condition indicated by profound and persistent social withdrawal (Cai et al. 2023). Etymologically, “hikikomori” combines the Japanese words *hiku* (“to retract”) and *komoru* (“to withdraw”, “seclude oneself” or “stay inside”), emphasizing the defining feature of the phenomenon: an individual's continued, voluntary withdrawal from social life. Such individuals typically stay at home and disengage from contact with others.

Identifying hikikomori requires meeting specific criteria, including profound social isolation in the home or ongoing social withdrawal lasting 6 months or more (Kato et al. 2020). First, it was mainly interpreted as largely culture‐specific to Japan, previous studies, however, have demonstrated that hikikomori symptoms are also frequent in other societies (e.g., in Oman (Al‐Sibani et al. 2023)). Previous bibliometric reviews has also shown a considerable increase in research activity in this area (Cai et al. 2023; Neoh et al. 2023).

Former research proposed a bio‐psycho‐socio‐cultural model to better understand the factors associated with hikikomori symptoms. For instance, depressive symptoms and social anxiety symptoms are associated with more severe hikikomori symptoms (Nonaka et al. 2025; Zhang et al. 2025). Other research showed that problematic social media use or gaming disorder are associated with hikikomori symptoms (Hajek, König, et al. 2026; Shah et al. 2024). Further research showed that being overwhelmed at work is associated with more severe hikikomori symptoms (Hajek, König, et al. 2026).

Beyond that, there is some evidence showing a positive association between higher loneliness levels (i.e., the difference between actual and desired social relationships) and more severe hikikomori symptoms (Hajek et al. 2024; Lyakina et al. 2023; Muris et al. 2025). To date, however, a systematic review plus meta‐analysis does not exist synthesizing (and quantifying) the existing evidence. Hence, we aimed to conduct a systematic review and meta‐analysis to synthesize the existing studies. The meta‐analysis specifically aimed to clarify the association between loneliness and hikikomori symptoms. We believe that our current work may add to our knowledge in this research field. Our work might determine gaps in the literature. This might guide future studies. Furthermore, the meta‐analysis may provide a better picture of the relationship between loneliness and hikikomori in comparison to evidence based on one specific sample. Moreover, a better understanding of this link may help to identify those at risk earlier and may help to provide more appropriate support (including the affected family members). It could also help to reduce the stigma surrounding individuals with severe hikikomori symptoms (by being perceived as less lazy (Majumder 2022) and instead as people with unmet social needs). Ultimately, a better understanding of the association between loneliness and hikikomori symptoms is also important because both can have deleterious effects (e.g., in terms of suicidal ideation or other important outcomes such as mortality) (Deason et al. 2025; Hajek et al. 2025; König and Hajek 2024).

## 2 Materials and Methods

This study was performed in line with the PRISMA (Preferred Reporting Items for Systematic Reviews and Meta‐Analyses) guidelines for systematic reviews and meta‐analyses (Page et al. 2021) and followed a protocol preregistered with PROSPERO (ID: CRD420261377402). Because of the restricted number of studies finally being included, a meta‐regression was not conducted.

Three well‐known databases (PubMed, CINAHL and Web of Science) were searched in May 2026. We also conducted a hand search. This included backward and forward citation searching. The search strategy and the selection of databases were established in close consultation with a librarian. More details of the search approach are provided in Supporting Information S1. Study selection was carried out independently by two reviewers (AH, HHK) in a two‐stage process: (1) title/abstract screening and (2) full‐text evaluation (plus hand search). Any disagreements were resolved through discussion between the two reviewers.

Studies were included if they (i) were observational, (ii) investigated the association between loneliness and hikikomori symptoms based on valid tools (e.g., 25‐item Hikikomori Questionnaire (HQ‐25) (Teo et al. 2018)), and (iii) were published in peer‐reviewed journals (in any language). We therefore excluded non‐observational studies and illness‐specific samples. To ensure an appropriate quality standard, non–peer‐reviewed studies were excluded. No restrictions were made with regard to the publication date or the location.

Before finalizing the inclusion criteria, we performed a pilot test (100 titles/abstracts). The inclusion criteria were not revised afterward. Data extraction was performed independently by two individuals (AH, HHK). Extracted information comprised the sample, tools used to measure loneliness and hikikomori symptoms, and sample characteristics, and the main findings. Any discrepancies between the two individuals were resolved through discussion.

Study quality was assessed by means of the frequently applied National Institutes of Health (NIH) quality assessment tool for observational cohort and cross‐sectional studies (National Heart and Blood Institute 2014). Two individuals (AH, HHK) independently conducted the assessments, and any discrepancies were resolved by means of discussion.

We conducted a random effects model (psychometric meta‐analysis) and reported corrected correlations that account for measurement error (see Wiernik and Dahlke 2020). The bootstrap method was used to replace missing reliability estimates. Some subgroup analyses were conducted (stratified by continent and by the tool used to quantify loneliness). We also analyzed the association of loneliness with the three subscales of the HQ‐25 (i.e., socialization subscale, isolation subscale, and emotional support subscale).

Consistent with common practice, we used the I^2^ statistic to quantify heterogeneity across studies. We applied the classification suggested by (Higgins et al. 2003): low (25%–50%), moderate (50%–75%), and high (≥ 75%). Guided by previous recommendations (Bosco et al. 2015; Brydges 2019; Gignac and Szodorai 2016; Paterson et al. 2016), we consider corrected correlations of 0.10, 0.30, and 0.50 as small, medium, and large, respectively. As the number of studies was small, we abstained from performing a funnel plot and calculate the (Egger et al. 1997) to check for publication bias. StataNow 19.5 (MP‐Parallel Edition; College Station, Texas, United States) was used for meta‐analysis.

## 3 Results

### 3.1 Study Overview

**FIGURE 1:**
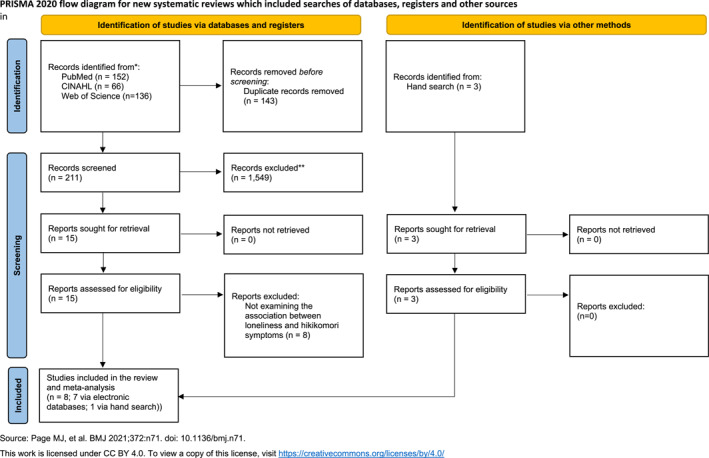
Flow‐chart.

Using PubMed, CINAHL and Web of Science, 211 hits were initially screened (title/abstract) after duplicate removal (see Figure 1 (Page et al. 2021)). In a second step, 15 full‐text articles were assessed. In sum, eight studies met our eligibility criteria and were finally included in this present work (Gündogmus et al. 2021; Hajek, König, et al. 2026; Hajek et al. 2024; Je et al. 2022; Kaya et al. 2023; Lyakina et al. 2023; Shah et al. 2024; Teo et al. 2018). Thereof, one study was included via hand search (Je et al. 2022).

We extracted key characteristics of the included studies (see Table 1). The date of publication varied from 2018 to 2026. Nearly all of such studies (except for (Teo et al. 2018)) were published from 2021 onwards. The studies used samples from Asia (e.g., Japan or Korea) (Je et al. 2022; Shah et al. 2024; Teo et al. 2018) and Europe (two studies each from Germany and Turkey and one study from Russia) (Gündogmus et al. 2021; Hajek, König, et al. 2026; Hajek et al. 2024; Kaya et al. 2023; Lyakina et al. 2023). All studies used cross‐sectional data (often collected via online surveys). Some studies used data from after the COVID‐19 pandemic, while others do not clearly specify when the data was collected. Many of the included studies have sample sizes of approximately 200–400 (except for the two German studies (Hajek, König, et al. 2026; Hajek et al. 2024) with samples in the mid‐four‐digit range). Several samples focused on students, whereas the two German studies focused on the general adult population using quota samples. This also goes well with the fact that the average age varies accordingly (mean ages of mid 20s in the studies focusing on students to about 47 years in the German studies focusing on the adult population). In many studies, roughly half of the respondents were women. All of the included studies used the HQ‐25 (or corresponding translations of this tool) to quantify hikikomori symptoms. The majority of the studies used the UCLA tool (mostly the 20‐item version) to operationalize loneliness, whereas the two German studies used the De Jong Gierveld (DJG) tool (6‐item version) to measure loneliness.

**TABLE 1:** Study overview and key findings.

Author (Year)	Country	Tools used to quantify loneliness and hikikomori symptoms	Sample and study type	Time of data collection	Sample size, age in total sample and sex ratio	Overall study quality
Gündogmus et al. (2021)	Turkey	HQ‐25 (Turkish version) UCLA (20‐item version)	Healthy participants (i.e., without any mental or physical illness)	Not reported	*n* = 343 Mean age: 33.2 years (SD: 13.6) Female: 46%	Fair
Hajek et al. (2024)	German	HQ‐25‐G (i.e., German version) DJG‐6	Individuals aged 18–74 years residing in Germany Quota‐based online survey	August and September 2024	*n* = 5000 Mean age: 46.9 years (SD: 15.3) Female: 50.8%	Good
Hajek, König, et al. (2026)	German	HQ‐25‐G (i.e., German version) DJG‐6	Individuals aged 18–74 years residing in Germany Quota‐based online survey	January 2025	*n* = 3270 Mean age: 47.0 years (SD: 15.3) Female: 50.4%	Good
Je et al. (2022)	Korea	HQ‐25 (Korean version). UCLA (20‐item version)	Individuals aged 13 years and older (online survey: 19 years and older) Online survey and in‐person survey	Not reported	*n* = 143 Mean age: not reported Female: Not reported	Fair
Kaya et al. (2023)	Turkey	HQ‐25 (Turkish version) UCLA (8 item version)	Nursing students	Not reported	*n* = 418 Mean age: not reported Female: 77.5%	Fair
Lyakina et al. (2023)	Russia	HQ‐25 (Russian version) UCLA (20 item version)	Student communities (various universities) Online survey	2021–2022	*n* = 451 Mean age: 23.2 (SD: 5.4) Female: 74.7%	Good
Shah et al. (2024)	United Arab Emirates and Oman in particular	HQ‐25 (English version) UCLA (20‐item version)	Individuals residing in the Middle East aged 18 yeras and older Participants from universities and social media (mixture of convenience and snowball sampling)	January to March 2024	*n* = 220 Mean age: 21.5 (3.3) Female: 46.4%	Good
Teo et al. (2018)	Japan	HQ‐25 UCLA (20‐item version)	Participants from clinical and community settings (Fukuoka, metropolitan area in southern Japan)	February 2014 to December 2016	*n* = 399 Mean age: 32 years (9.8) Female: 49.5%	Good

### 3.2 Meta‐Analysis

**FIGURE 2:**
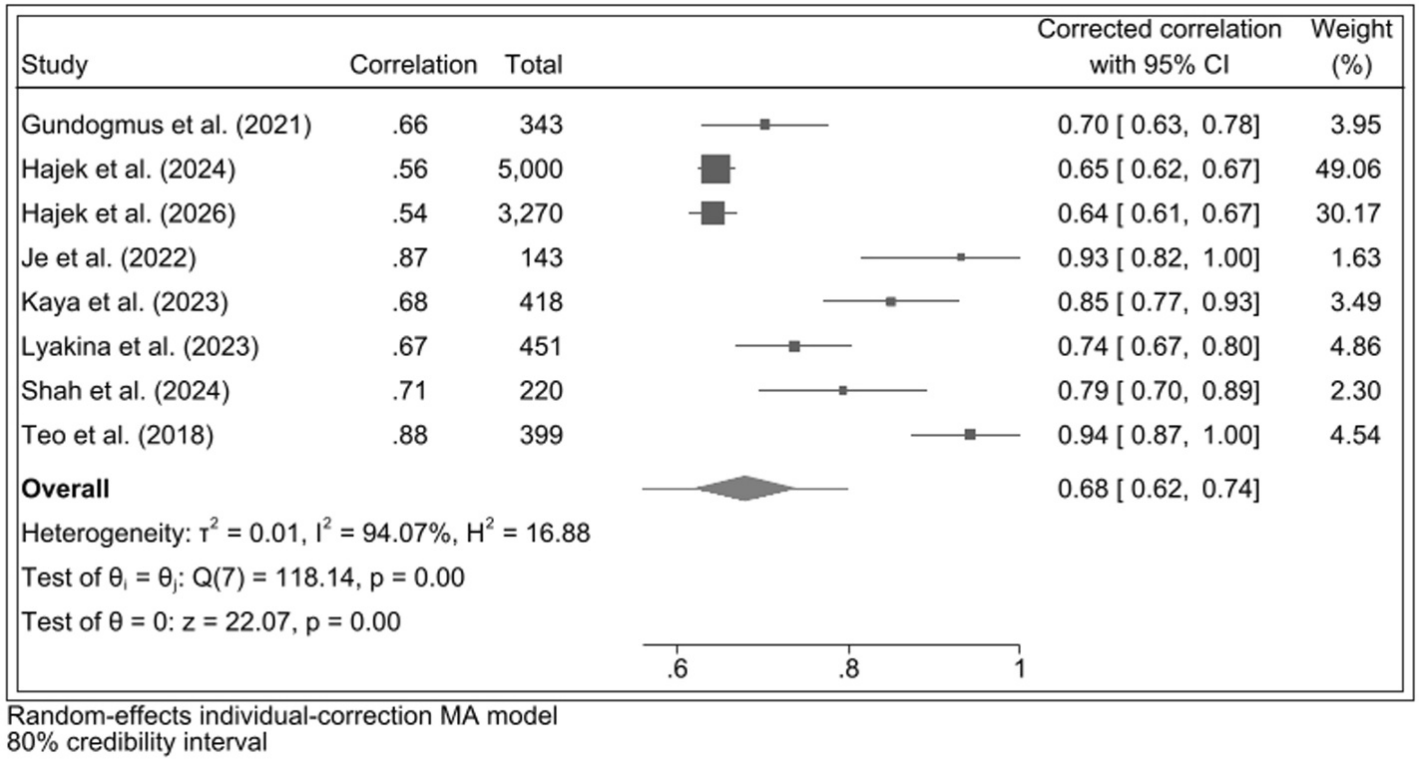
Meta‐analysis.

Based on all eight studies, the mean corrected correlation for the association between loneliness and hikikomori symptoms is 0.68 with a 95% CI of 0.62–0.74 (see Table 2; Figure 2). Five studies (see Table 2) also reported the association of loneliness with the subscales of the HQ‐25 (Gündogmus et al. 2021; Hajek, König, et al. 2026; Hajek et al. 2024; Lyakina et al. 2023; Shah et al. 2024). The mean corrected correlation for the association between loneliness and the socialization subscale was 0.54 (95% CI: 0.50 to 0.58), the mean corrected correlation for the association between loneliness and the isolation subscale was 0.62 (95% CI: 0.60 to 0.64), and the mean corrected correlation for the association between loneliness and the emotional support subscale was 0.71 (95% CI: 0.67 to 0.75). Notably, none of the studies provided sex‐or age‐stratified associations.

**TABLE 2:** Mean corrected correlation between loneliness and hikikomori symptoms (total sample).

Number of studies	Association between loneliness and hikikomori symptoms, 95% CI, I^2^ (%), *p*‐value	Association between loneliness and hikikomori symptoms (socialization subscale), 95% CI, I^2^ (%), *p*‐value	Association between loneliness and hikikomori symptoms (isolation subscale), 95% CI, I^2^ (%), *p*‐value	Association between loneliness and hikikomori symptoms (emotional support subscale), 95% CI, I^2^ (%), *p*‐value
8	0.68; 95% CI: 0.62 to 0.74 I^2^ = 94.1, *p* < 0.001			
5		0.54; 95% CI: 0.50 to 0.58 I^2^ = 75.8, *p* < 0.001	0.62 95% CI: 0.60 to 0.64 I^2^ = 0.0, *p* = 0.41	0.71 95% CI: 0.67 to 0.75 I^2^ = 81.1, *p* < 0.001

Stratified by subgroups (see Table 3), the mean corrected correlation for the association between loneliness and hikikomori symptoms was 0.66 (95% CI: 0.62 to 0.70) among European countries, whereas it was 0.90 (95% CI: 0.81 to 0.99) among Asian countries. The mean corrected correlation for the association between loneliness and hikikomori symptoms was 0.82 (95% CI: 0.73 to 0.90) among studies using the UCLA tool to quantify loneliness (95% CI: 0.62 to 0.70), whereas it was 0.64 (95% CI: 0.64 to 0.65) among studies using the DJG tool to measure loneliness.

**TABLE 3:** Mean corrected correlation between loneliness and hikikomori symptoms (subgroup analysis).

Stratified by	Subgroups	Number of studies	Association	95% CI	I^2^ (%), *p*‐value
Continent	Europe	5	0.66	0.62 to 0.70	87.6, *p* < 0.001
	Asia	3	0.90	0.81 to 0.99	68.3, *p* < 0.05
Tool used to quantify loneliness	UCLA tool	6	0.82	0.73 to 0.90	84.4, *p* < 0.001
	DJG tool	2	0.64	0.64 to 0.65	/

### 3.3 Quality Assessment/Risk of Bias Assessment

The overall evaluation of the quality (risk of bias assessment) is displayed in the last column of Table 1 (detailed assessment: see Supporting Information S1). Overall, five studies had a good quality, and the remaining studies had a fair quality. All included studies used psychometrically sound tools to examine loneliness and hikikomori symptoms. Frequent shortcomings were that no sample size justification was provided and the response rate was not given.

## 4 Discussion

The aim of our work was to synthesize the studies and to quantify the association between loneliness and hikikomori symptoms. Our meta‐analysis showed a strong, positive association between such factors. Such associations were particularly pronounced among Asian countries. Five of the studies were of good quality, and three were of fair quality. This is the first systematic review and meta‐analysis focusing on the link between loneliness and hikikomori symptoms and therefore clearly extends our current knowledge in the largely unexplored research field relating to hikikomori symptoms.

The general finding that higher loneliness levels are strongly associated with hikikomori symptoms is very plausible. Individuals scoring high in hikikomori symptoms often lead very reclusive lives (e.g., due to conflicts, pressure to perform, or experiences of bullying) (Guo 2022). As a result, this can severely limit contact with others and can be accompanied by feelings of diminished self‐worth and loneliness (Ranieri et al. 2015). Loneliness can also contribute to poor mental health (Mann et al. 2022), which can lead to withdrawal from society (Koyama et al. 2010).

The finding that loneliness was very strongly associated with hikikomori symptoms in Asian countries is worth noting. Previous research also cautiously assumed that “there is a very specific association between loneliness and hikikomori in East Asia” (Hajek et al. 2024) (p. 6). Potential reasons may refer to the fact that social pressure is often linked to societal roles (e.g., being productive, school performance) in Asian countries. Social withdrawal may be viewed as a deviation from such roles, which may reflect a burden for the family or loss of status. This may lead to feelings of shame, fear of being judged, concerns that relationships will end, and other negative consequences. To avoid such negative judgment, individuals may withdraw socially rather than seek help since talking openly about mental health may be less accepted. Such resulting feelings of loneliness may contribute to further avoidance and withdrawal behavior. Consequently, loneliness and hikikomori symptoms may be strongly correlated and reinforce each other.

Interestingly, our meta‐analysis also showed that loneliness was associated with the emotional support subscale of the HQ‐25 in particular. Individuals scoring high in this subscale may miss individuals they can trust and talk about important issues, and may have a lack of important relationships. Previous research has shown that such factors are closely associated with feelings of (emotional) loneliness (Salimi and Bozorgpour 2012). The fact that loneliness was associated with hikikomori symptoms particularly among studies using the UCLA tool may be explained by the fact that this tool more strongly refers to the feelings of not belonging to society or feeling excluded from society (which is obviously strongly linked to hikikomori symptoms), whereas the DJG tool mainly refers to the difference between actual and desired social relationships (see also (Hajek et al. 2024):).

The overall quality of the included studies was fair to good. A key strength of the included studies was that they used psychometrically sound tools to quantify the key variables. Several of the studies used hardly generalizable samples of students assessed at a single point in time.

Several research gaps were determined. The current studies were clearly restricted by using cross‐sectional data to examine the association between loneliness and hikikomori symptoms. There are arguments for both directions. For example, loneliness may contribute to feelings of shame or a lack of support, which may drive hikikomori symptoms. On the other side, extreme social withdrawal may reduce social contacts and activities over time, which may lead to loneliness. We believe that the relationship could be bidirectional. Future research is needed to test such directionalities over time (also in stratified analyses such as stratified by sex, age, or marital status). The included studies used data from Asia and Europe. Consequently, we encourage studies from other areas of the planet. Several of the studies used samples from specific groups (students in particular). Consequently, studies are required from general populations and other specific age brackets (e.g., adolescents or the oldest old) based on representative samples.

Our present work had some strengths and shortcomings. One should stress the fact that this is the first systematic review and meta‐analysis determining the association between loneliness and hikikomori symptoms. Our work, which satisfied the PRISMA guidelines, was registered (PROSPERO). Our search strategy was carefully coordinated with a librarian. We also performed a hand search. Important procedures were independently performed by two individuals. Due to the exclusive focus on peer‐reviewed studies, some studies may be excluded. However, we consider the peer‐review process to be of great importance for ensuring that studies meet a certain standard of quality.

In conclusion, there is a strong association between loneliness and hikikomori symptoms (particularly with the emotional support subscale and among Asian countries). Such knowledge is important to better characterize individuals at risk of having severe hikikomori symptoms. Future longitudinal studies are required to clarify the directionality between such factors. There is also a clear need for research in regions that have been neglected so far, such as North and South America, as well as Africa.

## Author Contributions

**André Hajek:** conceptualization, data curation, methodology, formal analysis, writing – original draft, writing – review and editing, project administration, visualization, **Hans‐Helmut König:** conceptualization, writing – review and editing, resources, visualization, supervision.

## Funding

The authors have nothing to report.

## Ethics Statement

The authors have nothing to report.

## Consent

The authors have nothing to report.

## Conflicts of Interest

The authors have no competing interests to declare that are relevant to the content of this article.

## Supporting information


Supporting Information S1

## Data Availability

All necessary information is provided in the tables and text. The corresponding author can be contacted for further details.
