# Supplementary material for: Association Between Loneliness and Hikikomori Symptoms. A Systematic Review and Meta‐Analysis
Source: Int J Methods Psychiatr Res. 2026 Aug 3;35(3):e70095. doi: 10.1002/mpr.70095 (PMC13433828; doi:10.1002/mpr.70095)
Supplement: Supplementary file 1 [file MPR-35-e70095-s001.docx]

Supplementary Table 1. Search strategy.

| Database | Search algorithm |
| --- | --- |
| Pubmed | ("social isolation"[MeSH Terms] OR "lonel*"[Title/Abstract]) AND "hikikomori"[Title/Abstract] |
| CINAHL | ((MH "social isolation+") OR (TI lonel* OR AB lonel*)) AND (TI hikikomori OR AB hikikomori) |
| Web of Science | ("social isolation" OR lonel*) AND hikikomori |

Supplementary Table 2. Quality assessment/risk of bias assessment.

| Paper author and date | Gundogmus et al. (2021) | Hajek et al. (2024) | Hajek et al. (2026) | Je et al. (2026) | Kaya et al. (2023) | Lyakina et al. (2023) | Shah et al. (2024) | Teo et al. (2018) |
| --- | --- | --- | --- | --- | --- | --- | --- | --- |
| 1. Was the research question or objective in this paper clearly stated? | Yes | Yes | Yes | Yes | Yes | Yes | Yes | Yes |
| 2. Was the study population clearly specified and defined? | No | Yes | Yes | No | No | Yes | No | Yes |
| 3. Was the participation rate of eligible persons at least 50%? | Not reported | Not reported | Not reported | Not reported | Not reported | Not reported | Not reported | Not reported |
| 4. Were all the subjects selected or recruited from the same or similar populations (including the same time period)? Were inclusion and exclusion criteria for being in the study prespecified and applied uniformly to all participants? | Yes | Yes | Yes | Yes | Yes | Yes | No | Yes |
| 5. Was a sample size justification, power description, or variance and effect estimates provided? | No | No | No | No | Yes | No | Yes | No |
| 6. For the analyses in this paper, were the exposure(s) of interest measured prior to the outcome(s) being measured? (if not prospective should be answered as 'no', even is exposure predated outcome) | No (cross-sectional) | No (cross-sectional) | No (cross-sectional) | No (cross-sectional) | No (cross-sectional) | No (cross-sectional) | No (cross-sectional) | No (cross-sectional) |
| 7. Was the timeframe sufficient so that one could reasonably expect to see an association between exposure and outcome if it existed? | No (cross-sectional) | No (cross-sectional) | No (cross-sectional) | No (cross-sectional) | No (cross-sectional) | No (cross-sectional) | No (cross-sectional) | No (cross-sectional) |
| 8. For exposures that can vary in amount or level, did the study examine different levels of the exposure as related to the outcome (e.g., categories of exposure, or exposure measured as continuous variable)? | Yes | Yes | Yes | Yes | Yes | Yes | Yes | Yes |
| 9. Were the exposure measures (independent variables) clearly defined, valid, reliable, and implemented consistently across all study participants? | Yes | Yes | Yes | Yes | Yes | Yes | Yes | Yes |
| 10. Was the exposure(s) assessed more than once over time? | No | No | No | No | No | No | No | No |
| 11. Were the outcome measures (dependent variables) clearly defined, valid, reliable, and implemented consistently across all study participants? | Yes | Yes | Yes | Yes | Yes | Yes | Yes | Yes |
| 12. Was loss to follow-up after baseline 20% or less? | Not applicable | Not applicable | Not applicable | Not applicable | Not applicable | Not applicable | Not applicable | Not applicable |
| 13. Were key potential confounding variables measured and adjusted statistically for their impact on the relationship between exposure(s) and outcome(s)? | No (validation study) | No (validation study) | Yes | No (validation study) | No (validation study) | No (validation study) | Yes | No (validation study) |
| Overall quality judgement | Fair | Good | Good | Fair | Fair | Good | Good | Good |
